# Bifidobacterium fermentum sp. nov. and Bifidobacterium aquikefiricola sp. nov., isolated from water kefir

**DOI:** 10.1099/ijsem.0.006549

**Published:** 2024-10-24

**Authors:** Samuel Breselge, Paolo Bellassi, Coral Barcenilla, Avelino Álvarez-Ordóñez, Lorenzo Morelli, Paul D. Cotter

**Affiliations:** 1 Teagasc Food Research Centre, Moorepark, Cork, Ireland; 2 APC Microbiome Ireland, Cork, Ireland; 3 Department for Sustainable Food Process-DiSTAS, Università Cattolica del Sacro Cuore, Via Bissolati, 74, 26100 Cremona, Italy; 4 Department of Food Hygiene and Technology, University of León, León, Spain; 5 VistaMilk, Cork, Ireland

**Keywords:** *Bifidobacterium*, metagenomics, polyphasic taxonomy, water kefir

## Abstract

Four strains, representing two novel *Bifidobacterium* species, were isolated from water kefir, a fermented beverage. 16S rRNA gene analysis suggested that the novel species share high identities (98.82–98.89%) with *Bifidobacterium aquikefiri* LMG 28769^T^. Complete genomes were assembled with a short- and long-read hybrid sequencing approach. In agreement with the 16S rRNA gene analysis, phylogenetics with 117 marker genes places the novel species closest to *B. aquikefiri* LMG 28769^T^ as well. The isolates have average nucleotide identity (ANI) scores ranging from 81.46 to 84.84% and digital DNA–DNA hybridization (dDDH) scores from 23.9 to 38.5% with the closest related species, as well as ANI scores between the proposed new species of 80.50%, indicating that the isolates represent two novel species. Matrix-assisted laser desorption/ionization-time of flight chemotaxonomic analysis supported the gene-based taxonomic placement. We propose the names *Bifidobacterium fermentum* sp. nov. and *Bifidobacterium aquikefiricola* sp. nov. for these novel species within the *Bifidobacterium* genus. The proposed type strain *B. fermentum* WK012_4_13^T^ (= LMG 33104^T^ = DSM 116073^T^; GenBank accession number GCF_041080835.1) has a genome size of 2.43 Mbp, with a G+C content of 56.00 mol%. The proposed type strain for *B. aquikefiricola* WK041_4_12^T^ (= LMG 33105^T^ = DSM 116074^T^; GenBank accession number GCF_041080795.1) has a genome size of 2.36 Mbp and a G+C content of 53.94 mol%. *B. fermentum* cells are Gram-positive staining, non-motile, non-spore-forming, fructose-6-phosphate phosphoketolase (F6PPK)-positive, catalase- and oxidase-negative and bacillary club shaped. *B. aquikefiricola* cells are Gram-positive staining, non-motile, non-spore-forming, F6PPK-positive, catalase- and oxidase-negative and square rod shaped.

## Introduction

Fermented foods and beverages are consumed worldwide [1]. Over the years, researchers have explored the beneficial properties of these foods and beverages for the human body [2]. Water kefir, a beverage with a complex microbial community, is distinguished by its effervescence and distinctive flavour. The water kefir fermentation converts sugars into various metabolic by-products, including organic acids, carbon dioxide, low levels of alcohol and various flavour compounds [3]. This transformation, which contributes to the overall flavour and nutritional profile of the beverage, is facilitated by the intricate network of micro-organisms within the inoculum [4]. Water kefir has also gained attention due to culture-independent studies, highlighting that the beverage is a potential reservoir for novel species [5], and on the basis of recent investigations, we predicted the existence of two novel *Bifidobacterium* species in water kefir based on metagenome-assembled genomes (MAGs) obtained from shotgun sequencing data (publication in preparation). Here, we present two novel *Bifidobacterium* species isolated from water kefir. Although the cultivation and isolation of bifidobacteria frequently pose considerable challenges due to their distinct growth requirements [6], the novel species, in contrast to the typical mesophilic and obligate anaerobic nature of bifidobacteria, exhibit aerotolerance and demonstrate optimal growth at a relatively low temperature of 30 °C.

## Methods

### Isolation from water kefir

As part of a microbial diversity analysis of water kefir samples obtained from different countries, two novel species belonging to the genus *Bifidobacterium* were identified in metagenomic sequencing data, including the four strains under study. Plates of De Man–Rogosa–Sharpeagar (MRS; Oxoid, England, UK) supplemented with 0.05 g l^−1^ mupirocin, 0.005 g l^−1^ amphotericin B and 0.5 g l^−1^ cysteine were used as selective media to isolate colonies belonging to the *Bifidobacterium* genus (medium modified from Ref. [7]). The plates were incubated under anaerobic conditions using Anaerocult A gas packs (Merck, Germany). Single colonies were randomly picked, and *Bifidobacterium* genus-specific PCRs were used to confirm that the isolates belonged to *Bifidobacterium* genus [8]. Colonies belonging to the genus *Bifidobacterium* were subjected to 16S rRNA gene amplification, and the PCR products were subjected to Sanger sequencing (Eurofins Scientific, Luxembourg). The type strain *Bifidobacterium aquikefiri* LMG 28769^T^ was purchased from the BCCM/LMG Bacteria Collection and analysed with the newly isolated strains from this study.

### DNA extraction, genome sequencing and genome assembly

Genomic DNA was extracted using the GenElute Bacterial Genomic DNA Kit (Sigma-Aldrich, USA). Four strains, representing the two novel species, were fully genome sequenced using a hybrid approach. Illumina sequencing was carried out according to the manufacturer’s instructions on a NextSeq 2000 P1 chip with a read length of 2×150 bp. Illumina read quality control was performed using MetaWRAP (v1.3.2) [9], with hg38 for host removal. Oxford Nanopore Technologies (ONT) sequencing was carried out using a native barcoding kit and an R10.4.1 flow cell, according to the manufacturer’s instructions, at 400 bp s^−1^. Super accuracy (SUP) mode basecalling and adapter trimming were performed using Guppy (v6.3.8; https://community.nanoporetech.com). ONT read quality control was performed using Filtlong (v0.2.0; https://github.com/rrwick/Filtlong), removing reads less than 1000 bp long and 10% of the reads with the lowest quality. Draft genomes were assembled using Flye (v2.9 [10], followed by long-read polishing with Medaka (v1.7.2; https://github.com/nanoporetech/medaka). Short-read polishing was performed using Polypolish (v0.5.0) [11]. Additional small plasmids were assembled using Unicycler (v0.4.7) [12]. Assembly quality was checked using CheckM (v1.0.18) [13] and CheckM2 (v0.1.3) [14]. Genome assemblies, including the G+C content, were assessed using QUAST (v5.1.0) [15]. Plasmids were confirmed using PLATON (v1.6) [16]. Genomes were annotated using PGAP (v 6.5) [17]. Average nucleotide identity (ANI) scores were calculated using FastANI (v1.32) [18] against all NCBI RefSeq and GenBank genomes of the genus *Bifidobacterium* (as of 11 June 2024). A phylogenetic tree was inferred for the novel species, with the GTDB-Tk curated set of 115 *Bifidobacterium* genomes derived from type strains and reference strains, along with one outgroup, using the *de novo* workflow in GTDB-Tk (v2.1.1) [19]. The *de novo* workflow used 117 marker genes and WAG+GAMMA models to infer the tree. The CGE webserver (https://cge.food.dtu.dk/services/ResFinder-EFSA/) [20] was used to scan the genomes for potential antimicrobial resistance genes. No resistances were predicted in any of the genomes.

### 16S rRNA phylogenetic tree

blastn analysis was conducted with the nucleotide collection in GenBank (https://blast.ncbi.nlm.nih.gov/Blast.cgi) using prokaryotic databases of 16S rRNA genes obtained from all type strains to determine sequence similarities. The phylogenetic study used 101 species attributed to the genus *Bifidobacterium* from the List of Prokaryotic Names with Standing in Nomenclature (LPSN). First, multiple sequence alignments were performed using clustal W [21]. Gap-containing regions were removed. Maximum-likelihood phylogenetic trees were generated from the aligned, gap-free sequences of ~900 base pairs. A bootstrap analysis of 1000 replicates was performed using mega 11 [22]. In addition, the ‘Find the best DNA/Protein (ML) models’ function, based on the value of the Bayesian information criterion, was used to determine the most appropriate nucleotide substitution model for reconstructing the phylogenetic tree [23].

### Phenotypic characteristics

Scanning electron microscopy (SEM) was used to evaluate the cell morphology (size and shape) of the strains under study. Fresh cultures were grown in MRS broth supplemented with 0.5 g l^−1^ cysteine (MRScys), pelleted at 4000 ***g*** for 5 min, washed in PBS and prepared for SEM observation by performing an overnight fixation in 2.5% glutaraldehyde (Sigma-Aldrich) in a 100 mM sodium cacodylate (pH 7.3; Sigma-Aldrich) solution, followed by dehydration in a 10% step increase gradient of aqueous ethanol solutions, from 30 to 100% (v/v) over a period of 4 days. The ethanol was replaced with hexamethyldisilazane (HMDS; Sigma-Aldrich) by immersing the samples in a 50 : 50% HMDS:ethanol solution for 1 h, followed by 100% HMDS for another hour. The HMDS was left to evaporate completely. Samples were fixed to the sample holders using carbon tape and sputter coated with gold under vacuum, using an Emitech K575X sputter coater (Quorum Technologies, UK). Zeiss Supra 40VP, equipped with a third-generation Gemini column (ZEISS, Germany), using a secondary electron detector, was used for SEM imaging. The resulting images were measured in ImageJ (v1.53) to assess the average cell size [24]. Growth capacity was assessed at 4, 15, 21, 30, 37 and 45 °C, with pH levels of 3, 4, 5, 6, 7, 8, 9 and 10 and NaCl concentrations of 0, 1, 3, 5, 7 and 10% under anaerobic conditions using MRScys by measuring OD_600_ after 72 h of incubation. The inoculum for each test was prepared by starting with an overnight culture grown in MRScys broth under anaerobic conditions at 30 °C; then, the overnight culture was adjusted to an OD_600_ of 0.5 and the inoculum was used at 1%. The maximum growth capacity (optimum) was considered to be the maximum OD_600_ achieved under all conditions tested. The maximum growth rate was only assessed at 20, 30 and 37 °C and evaluated by measuring OD_600_ every 2 h for 72 h. The efficiency of growth under aerobic and anaerobic conditions was tested for 72 h at 30 °C in MRScys broth under agitation to maximize oxygen exchange and in static mode using anaerobic jars under anaerobic conditions [25]. Growth under different conditions was determined on the basis of optical density: good growth OD_600_ ≥0.3, weak growth 0.1 ≤ OD_600_ ≤ 0.3 and no growth OD_600_ ≤0.1. Additionally, the ability to generate bacterial colonies under aerobic conditions was tested. Oxidase activity assays were carried out using the Bactident Oxidase kit (Merck Millipore), and catalase activity was verified by putting a small amount of strain biomass into two drops of 3% H_2_O_2_, according to Reiner [26]. Motility was assessed in accordance with the method described by Tittsler *et al.* [27] using MRScys supplemented with 0.3% agar. Fructose-6-phosphate phosphoketolase (F6PPK) was assessed using a colorimetric method [28]. The ability to ferment different substrates was assessed through API 50 CHL (bioMérieux, France). For this, 2 McFarland of pure culture, resuspended in an API 50 CHL medium flask, was added to each API 50 CHL strip. The results were read out after 72 h. Enzymatic activities were evaluated using the API ZYM (bioMérieux) following the manufacturer’s instructions. For this, 4 McFarland cell suspensions in 0.85% NaCl were inoculated into API ZYM strips. After 4 h, the results were read out.

### Chemotaxonomic analysis

For matrix-assisted laser desorption/ionization-time of flight (MALDI-TOF) mass spectrometry analysis, the ethanol/formic acid extraction procedure was used following Bruker’s guidelines. Briefly, the novel strains and the reference strain *B. aquikefiri* LMG 28769^T^ were grown anaerobically in MRS agar (VWR, Belgium) supplemented with 0.05% cysteine–HCl at 30 °C for 72 h. A single colony was resuspended in an Eppendorf tube with 300 µl sterile distilled water, followed by the addition of 900 µl of pure ethanol. The suspension was centrifuged at 21 036 ***g*** for 2 min, and the supernatant was discarded. To the dry pellet, 10 µl of 70% formic acid and 10 µl of acetonitrile were added and mixed well. The suspension was centrifuged again under the same conditions, and 1 µl of the supernatant was spotted onto a target polished steel BC plate (Bruker, Germany). After air-drying, 1 µl of the α-cyano-4-hydroxycinnamic acid matrix solution (Bruker), prepared following the manufacturer’s instructions in acetonitrile/water/trifluoroacetic acid (50 : 47.5 :  2.5 v/v), was added. Once dried at room temperature, the plate, with each of the samples and a Bacterial Test Standard (BTS, Bruker), was read in a MALDI-TOF MS microflex LRF mass spectrometer using flexControl v. 3.4 and MBT Compass v. 4.1.90. MALDI Biotyper Compass Explorer v. 4.1.90 was employed to compare the profiles of the novel species with that of the nearest neighbour. A main spectra profile (MSP) dendrogram was represented, including 25 spectra profiles of *Bifidobacterium* from the Bruker taxonomy database and the spectrum of the reference strain *B. aquikefiri* LMG 28769^T^. Finally, flexAnalysis v. 3.4 was used to represent the spectra profiles of *B. aquikefiri* LMG 28769^T^, WK012_4_13^T^ and WK041_4_12^T^.

## Results and discussion

We recently investigated 69 water kefir grains from 21 different countries with shotgun metagenomics (publication in preparation). MAG reconstruction from the shotgun metagenomics data suggested the presence of two candidate novel *Bifidobacterium* species. The first candidate new species (including the later isolate WK012_4_13^T^) was predicted to be present in 12 water kefirs, and the second species (the later isolate WK041_4_12^T^) was predicted to be present in only one water kefir. Four water kefirs were chosen and used to successfully isolate the predicted new species, resulting in four strains belonging to the two candidate new species.

### Genomic features and phylogeny

**Fig. 1.**
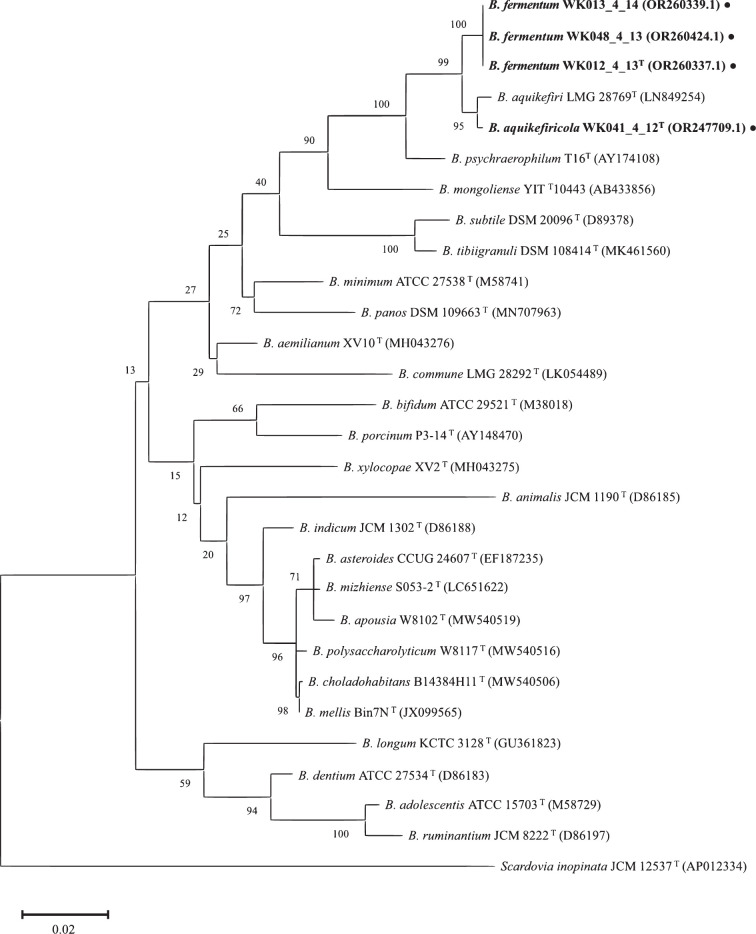
Phylogenetic tree of strains WK012_4_13^T^, WK013_4_14, WK048_4_13 and WK041_4_12^T^ and its relationship to other closely related species of the genus *Bifidobacterium*. The Tamura–Nei substitution model was used for this analysis. The tree nodes show the bootstrap values (>50%) obtained from 1000 replications. The scale bar, labelled 0.02, represents the number of substitutions per site. To ensure accuracy, all locations with gaps and missing data were excluded (complete deletion option). The analysis included a total of 29 nucleotide sequences, with 1331 positions in the final dataset. The sequence of *Scardovia inopinata* JCM 12537^T^ was used as an outgroup. For an extended version of this tree, please refer Fig. S1 (available in the online version of this article).

The pairwise similarity analysis of the 16S rRNA gene, performed with blast, revealed that the greatest sequence similarities were with *B. aquikefiri* LMG 28769^T^ with a similarity percentage ranging from 98.82 to 98.89%. In accordance with Riesco and Trujillo [29], who proposed a workflow for taxonomic classification based on 16S rRNA gene and genome sequences, all strains were subjected to the computation of the overall genome correlation index (OGRI). The phylogenetic tree, based on the 16S rRNA gene (Fig. 1), revealed that all strains formed a cluster together with *B. aquikefiri* LMG 28769^T^. Notably, strains WK048_4_13, WK013_4_14 and WK012_4_13^T^ formed a distinct cluster, confirming their membership in the same species, while WK041_4_12^T^ appeared to be more closely related to *B. aquikefiri* LMG 28769^T^. The high bootstrap values, exceeding 90, indicate a high level of confidence in the phylogenetic branches that encompass the studied strains in a resampled dataset. Based on the phylogenetic analyses, *B. aquikefiri* LMG 28769^T^ was chosen as the closely related species for further phenotypic comparisons.

The hybrid sequencing approach, with coverage ranging from 162× to 441× for Illumina reads and 410× to 976× for ONT reads, allowed the assembly of complete, circular genomes for all strains. Complete and circular plasmids were detected in WK012_4_13 ^T^ and WK048_4_13, but not in WK013_4_14 and WK041_4_12^T^ (Table 1). The genome sizes (excluding plasmids) were 2.43, 2.58, 2.65 and 2.36 Mbp for WK012_4_13^T^, WK013_4_14, WK048_4_13 and WK041_4_12^T^, respectively. The G+C content ranged between 55.82 and 56.00 mol% for WK012_4_13^T^, WK013_4_14 and WK048_4_13 and it is 53.94 mol% for WK041_4_12^T^*,* slightly higher than that in *B. aquikefiri* (52.0 mol%). The genome assemblies were used for OGRI calculation. WK012_4_13^T^ shares the highest ANI scores with *B. aquikefiri*, WK013_4_14 shares the highest ANI scores with *Bifidobacterium psychraerophilum*, WK048_4_13 shares the highest ANI scores with *B. psychraerophilum* and WK041_4_12^T^ shares the highest ANI scores with *B. aquikefiri* (Table 2). WK012_4_13^T^, WK013_4_14 and WK048_4_13 share ANI scores >99.9%, which classifies them as the same species according to Riesco and Trujillo [29]. WK041_4_12^T^ shares ANI scores of 81.69–80.50% with WK012_4_13^T^, WK013_4_14 and WK048_4_13, classifying it as a separate species. The digital DNA–DNA hybridization (dDDH) scores (see Table 1) also agree with WK012_4_13^T^, WK013_4_14, WK048_4_13 and WK041_4_12^T^ being classified as two novel species, as the dDDH scores are between 23.9 and 38.5%, well below the recommended value of ≤70% dDDH for a novel species. Additional genome features are given in Table 1.

**Table 1.** Genomic features summary table of strains belonging to *Bifidobacterium fermentum*, *B. aquikefiricola* and the type strain of the closely related species within the *Bifidobacterium* genus

Strain	*B. fermentum* WK012_4_13^T^	*B. fermentum* WK013_4_14	*B. fermentum* WK048_4_13	*B. aquikefiricola* WK041_4_12^T^	*B. aquikefiri*LMG 28769^T^
Source country	Italy	Germany	France	Singapore	Belgium
Genome accession nr.	GCF_041080835.1	GCF_041080825.1	GCF_041080805.1	GCF_041080795.1	GCF_002259795.1
16S rRNA gene accession nr.	OR260337	OR260339	OR260424	OR247709	LN849254
Genome size (Mbp) (including plasmids)	2.47	2.58	2.74	2.36	2.41
G+C content (mol%)	56.00	55.87	55.82	53.94	52.0
Contigs	2*	1*	7*	1*	18
Plasmid length	42795 bp		41 068 + 27443 + 5422 + 3102 + 2836 + 2572 bp		176599 + 34114 + 6671 bp
CDS (PGAP; total)	1954	2034	2207	1952	1992
tRNA (PGAP)	46	46	46	45	48
CheckM completeness (%)	98.72	98.72	98.27	98.78	98.83
CheckM contamination (%)	2.88	3.11	3.11	3.33	2.97
CheckM2 completeness (%)	98.73	98.69	97.84	99.17	99.53
CheckM2 contamination (%)	0.30	0.61	0.76	0.66	0.53
ANI to WK012_4_13^T^ (%)	100.00	99.95	99.96	80.50	
dDDH	23.9% *B. aquikefiri*LMG 28769^T^	38.5% *B. psychraerophilum*DSM 22366^T^	38.3% *B. psychraerophilum*DSM 22366^T^	37.7% *B. minimum*LMG 11592	
16S identity to best NCBI match (DB: rRNA_typestrains/16S_ribosomal_RNA)	98.82% *B. aquikefiri*LMG 28769^T^	98.82% *B. aquikefiri*LMG 28769^T^	98.82% *B. aquikefiri*LMG 28769^T^	98.89% *B. aquikefiri*LMG 28769^T^	

*aAll circular.

**Table 2.** WK012_4_13^T^, WK013_4_14, WK048_4_13 and WK041_4_12^T^ genome assemblies show the highest OGRI values (% ANI) with *B. aquikefiri* LMG 28769^T^, *B. psychraerophilum* DSM 22366^T^ and *Bifidobacterium crudilactis* LMG 23609^T^

	*B. aquikefiri*LMG 28769**^T^**	*B. psychraerophilum*DSM 22366**^T^**	*B. crudilactis*LMG 23609**^T^**
***B. fermentum* WK012_4_13^T^**	81.46	78.11	78.21
***B. fermentum* WK013_4_14**	80.95	82.31	78.75
***B. fermentum* WK048_4_13**	81.77	82.53	79.07
***B. aquikefiricola* WK041_4_12^T^**	84.84	81.72	<70

**Fig. 2.**
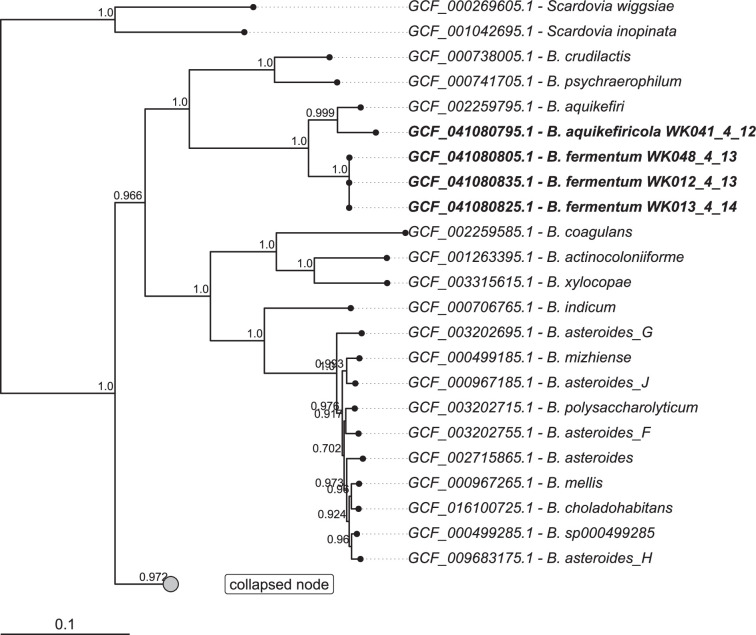
The phylogenetic tree was inferred using the *de novo* workflow with curated bifidobacteria genomes and 117 marker genes in GTDB-Tk [7] using the WAG+GAMMA model. *S. inopinata* JCM 12537^T^ and *Scardovia wiggsie* F0424 were used as outgroups. The collapsed node contains an additional 98 leaves. The full tree, with expanded node *g__Bifidobacterium,* can be found in Fig. S2.

The maximum-likelihood tree based on the GBTD-Tk tool showed a unique cluster composed of the four strains under study (Fig. 2). Marker-based phylogenetics places WK041_4_12^T^ closest to *B. aquikefiri* and WK012_4_13^T^, WK013_4_14 and WK048_4_13 on the same subclade. The sub-clusters confirmed the distribution observed in the phylogenetic tree obtained with the 16S rRNA gene. This result confirmed the choice of *B. aquikefiri* as the closely related species for the two new candidate species.

### Phenotypic features

**Fig. 3.**
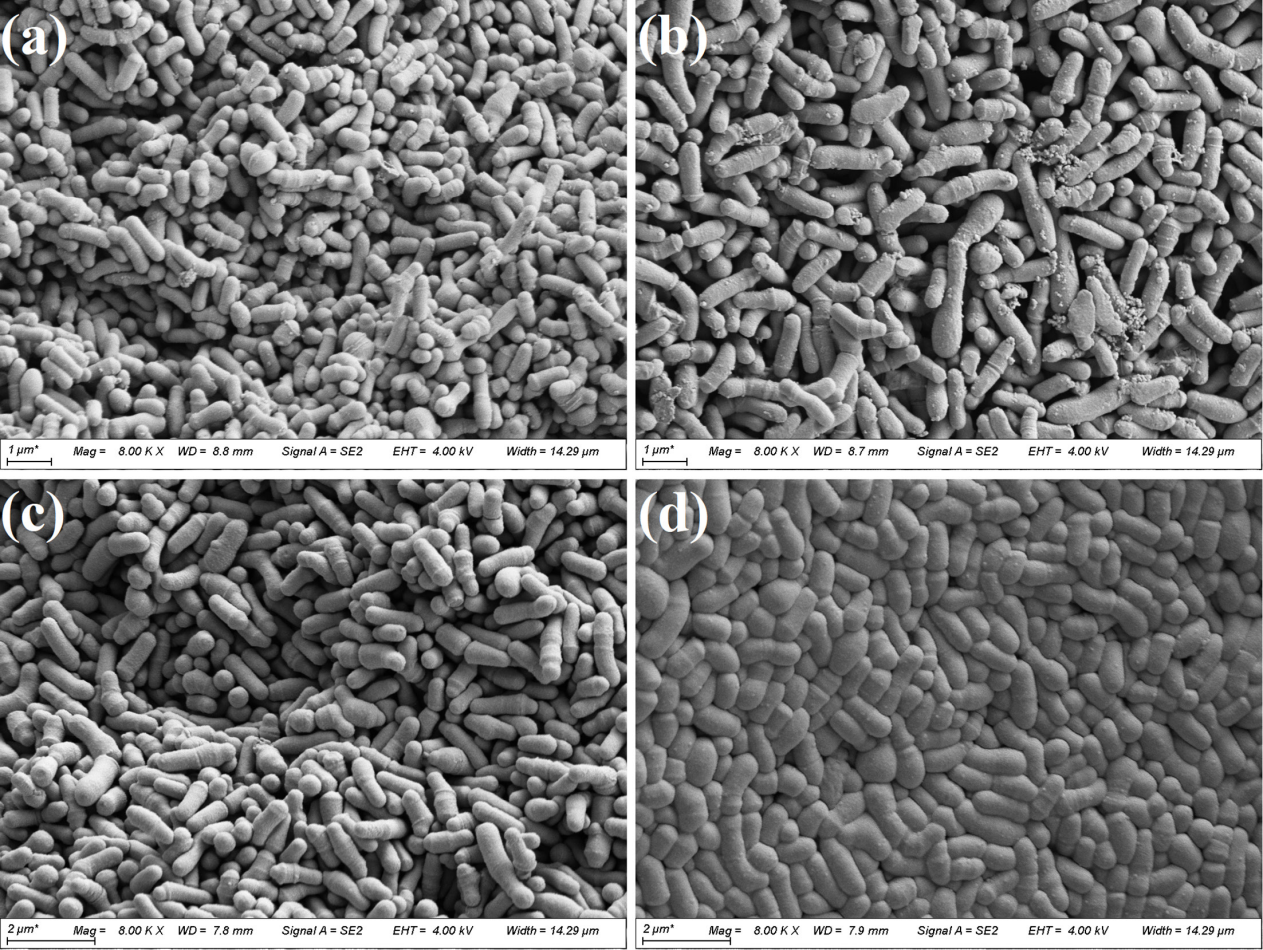
Image obtained by SEM showing the morphology and cell size of the four strains after incubation in MRScys at 30 °C for 48 h: (**a**) WK048_4_13, (**b**) WK013_4_14, (**c**) WK012_4_13^T^ and (**d**) WK041_4_12^T^.

SEM image analysis revealed that the cells of strains WK048_4_13, WK013_4_14 and WK012_4_13^T^ had a club-shaped and bacillary shape with average lengths of 1.23±0.17 µm, 1.38±0.25 µm and 1.05±0.18 µm and widths of 0.38±0.04 µm, 0.40±0.06 µm and 0.33±0.05 µm, respectively. Strain WK041_4_12^T^ had a square rod shape with an average length of 1.18±0.23 µm and an average width of 0.48±0.08 µm (Fig. 3). All strains were Gram-positive staining. The colonies were white and spherical after 48 h of incubation on MRScys agar. *B. aquikefiri* LMG 28769^T^ was chosen for comparison in the phenotypic tests. Strains WK048_4_13 and WK012_4_13^T^ were able to grow between 20 and 37 °C, while WK013_4_14 showed weak growth at 15 °C. WK041_4_12^T^ was able to grow between 15 and 37 °C, while *B. aquikefiri* LMG 28769^T^ also showed weak growth at 4 and 45°C. The optimum growth temperature based on the maximum growth capacity was 30 °C for all strains studied. Considering the maximum growth rate, strain WK041_4_12^T^ showed the maximum growth rate at 37 °C. All strains grew in a pH range between 4 and 8, except for strain WK048_4_13, which showed weak growth at pH 9. All strains had a higher maximum growth capacity at pH 6 (Table 3). The growth in the presence of increasing NaCl concentrations showed that strains WK048_4_13, WK041_4_12^T^ and WK012_4_13^T^ grow in a range of 0–3%, while WK013_4_14 and *B. aquikefiri* LMG 28769^T^ showed a growth range of 0–1%. The strains tested were able to grow well under aerobic conditions in MRScys agar and broth, including the reference strain * B. aquikefiri* LMG 28769^T^, except strain WK012_4_13^T^, which showed weak growth. Species of the genus *Bifidobacterium*, isolated from water kefir, such as the species *B. aquikefiri* [30], *Bifidobacterium tibigranuli* [31] and *B. psychraerophilum*, demonstrated the ability to tolerate aerobic environments [28]. The backslopping process of water kefir grains creates aerobic conditions, making it essential for the colonization of water kefir grains to be more resistant to oxidative stress.

**Table 3.** Differential characteristics of strains *B. fermentum* WK012_4_1^T^, WK013_4_14, WK048_4_13, *B. aquikefiricola* WK041_4_12^T^ and *B. aquikefiri* LMG 28769^T^, the closest related species within the *Bifidobacterium* genus* Strains: 1, WK012_4_13^T^; 2, WK013_4_14; 3, WK048_4_13; 4, WK041_4_12^T^; 5, *B. aquikefiri* LMG 28769^T^. Data were generated in this study, unless otherwise stated. Symbols: +, positive; –, negative; w, weak positive reaction; nd, not determined.

Characteristics	1	2	3	4	5
Temperature range for growth (°C)	20–37	15^ a^–37	20–37	15–37	4^ a^–45^a^
Optimum temperature (°C)	30	30	30	30(37)†	30
pH range for growth	4–9^a^	4–8	4–8	4–8	4–8
Optimum pH	6	6	6	6	6
NaCl range of growth (w/v, %)	0–3^b^	0–1	0–3^a^	0–3^b^	0–1
Enzymatic activity (API ZYM):					
*α*-Glucosidase	+	+	+	+	+
Esterase (C4), naphthol-AS-BI-phosphohydrolase, alkaline phosphatase and trypsin	–	–	–	–	w
Valine arylamidase	–	–	–	w	w
*β*-Galactosidase	+	+	+	–	+
*β*-Glucosidase and leucine arylamidase	w	w	w	w	+
*N*-Acetyl-*β*-glucosaminidase	–	–	–	+	–
Cystine arylamidase	w	w	w	w	w
Acid phosphatase	w	w	+	w	+
*α*-Galactosidase	w	w	+	–	+
Acid production from (API 50CH):					
d-Arabinose and d-Xylose	+	+	+	–	–
l-Arabinose	–	–	–	–	+
d-Galactose and potassium gluconate	w	w	w	–	+
d-Glucose	w	+	w	+	+
d-Mannose and d-saccharose	+	+	+	–	+
Dulcitol	–	–	–	+	–
Methyl-*α*-d-glucopyranoside	+	+	+	w	+
*N*-Acetylglucosamine and potassium 5-ketogluconate	–	–	–	–	w
Amygdalin, arbutin, salicin and d-cellobiose	–	+	–	–	–
Gentiobiose	w	w	–	–	–
d-Turanose	w	–	–	–	+
d-Lyxose	w	w	w	–	–

*All strains showed no motility, catalase or oxidase activity. All strains were lipase (C14), *α*-chymotrypsin, ß*β*-glucuronidase, *α*-mannosidase, *α*-fucosidase and esterase lipase (C8) negative. All strains showed no acid production with glycerol, erythritol, l-xylose, d-adonitol, methyl-*β*-d-xylopyranoside, l-sorbose, l-rhamnose, inositol, d-mannitol, d-sorbitol, methyl-*α*-d-mannopyranoside, d-trehalose, inulin, d-melezitose, starch, glycogen, xylitol, d-tagatose, d-fucose, l-fucose, d-arabitol, l-arabitol, potassium 2-ketogluconate, d-lactose**.** All strains showed acid production with d-ribose, d-fructose, esculin ferric citrate, d-maltose, d-melibiose, and d-raffinose**.**

†Resulting optimum based on maximum growth rate.

aWeak growth.

bGood growth.

All strains tested negative in catalase, oxidase and motility tests. F6PPK was tested as a taxonomic marker commonly used to identify the genus *Bifidobacterium*. All strains showed F6PPK activity. Based on the API 50 CHL strip, it was possible to identify the ability to produce acid with different carbohydrates and derivatives. All strains, including the reference strain * B. aquikefiri* LMG 28769^T^, were able to produce acid from d-ribose, d-fructose, aesculin ferric citrate, d-maltose, d-melibiose and d-raffinose, but not from glycerol, erythritol, l-xylose, d-adonitol, methyl-*β*-d-xylopyranoside, l-sorbose, l-ramnose, inositol, d-mannitol, d-sorbitol, methyl-*α*-d-mannopyranoside, d-trehalose, inulin, d-melezitose, starch, glycogen, xylitol, d-tagatose, d-fucose, l-fucose, d-arabitol, l-arabitol, potassium 2-ketoglucose and d-lactose.

Table 3 shows the distinguishing characteristics between strains WK048_4_13, WK013_4_14, WK012_4_13^T^, WK041_4_12^T^ and *B. aquikefiri* LMG 28769^T^. In particular, strain WK041_4_12^T^ produced acid from dulcitol, but not from d-arabinose, d-xylose, d-galactose, d-mannose, d-saccharose, potassium gluconate and d-lyxose compared to strains WK048_4_13, WK013_4_14 and WK012_4_13^T^. The carbohydrate fermentation profile observed in strain WK013_4_14 shows the ability to produce acid, unlike the other strains, using amygdalin, arbutin, salicin and d-cellobiose. The latter difference in carbohydrate fermentation in strain WK013_4_14 suggested a strain-specific ability to ferment carbohydrates and derivatives.

In addition, the API ZYM system was used to test the enzymatic production of the strains. Strain WK041_4_12^T^ exclusively produced *N*-acetyl-β-glucosaminidase and weak valine arylamidase. In contrast, it did not produce α-galactosidase and β-galactosidase. The carbohydrate fermentation profile of *B. aquikefiri* LMG 28769^T^ is in agreement with the results obtained by Laureys *et al.* [30], except for d-mannitol, amygdalin, aesculin ferric citrate, gentiobiose and potassium 5-ketogluconate.

### Chemotaxonomic analysis

**Fig. 4.**
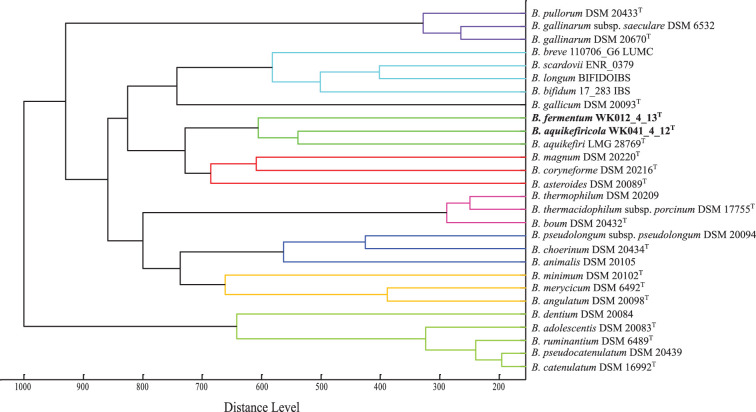
MSP-based dendrogram of the two novel species, the closely related reference strain *Bi. aquikefiri* LMG 28769^T^, and 25 strains from the Bruker taxonomy library representing the genus *Bifidobacterium*. The distances are normalized to a maximum value of 1000 and correspond to the relative similarity of MS spectra.

The two mass spectra profiles from the candidate novel species were compared with that of *B. aquikefiri* LMG 28769^T^ (Fig. S3). In addition, they were evaluated with spectra from other 25 known *Bifidobacterium* spp. strains included in the Bruker Taxonomy database (Fig. 4). The proposed novel species did not match any of the species found in the database, suggesting possible new species. In the MSP dendrogram, *B. aquikefiri* LMG 28769^T^ is the closest species based on the distance matrix to WK012_4_13^T^ and WK041_4_12^T^, especially to the latter one. Their clustering at a distance level of >500 underlines the possibility of being novel species. These MALDI-TOF results are thus in agreement with the results obtained in the 16S rRNA and GTDB-TK phylogenetic trees.

## Description of *B. fermentum* sp. nov.

*B. fermentum* (fer.men′tum. L. neut. n. *fermentum*, that which causes fermentation).

The cells are Gram-positive staining, non-motile, non-spore-forming, F6PPK-positive, catalase- and oxidase-negative, bacillary club shape with an average length of 1.23±0.17 µm. The colonies are white and spherical after 48 h of incubation in MRScys. A good growth occurs under anaerobic conditions, in the temperature interval of 20–37 °C, the pH range of 4–8 and NaCl range of 0–1%. Optimal conditions for growth occur at 30 °C and a pH value of 6. Strong growth occurs under anaerobic conditions for all three strains analysed, and aerophilic conditions for two of the three strains analysed. WK012_4_13^T^ showed weak growth under aerobic conditions. Fermentation occurred from d-ribose, d-fructose, aesculin ferric citrate, d-maltose, d-melibiose, d-raffinose, d-arabinose, d-xylose, d-mannose, d-saccharose and methyl-*α*-d-glucopyranoside, but not from glycerol, erythritol, l-xylose, d-adonitol, methyl-*β*-d-xylopyranoside, l-sorbose, l-rhamnose, inositol, d-mannitol, d-sorbitol, methyl-*α*-d-mannopyranoside, d-trehalose, inulin, d-melezitose, starch, glycogen, xylitol, d-tagatose, d-fucose, l-fucose, d-arabitol, l-arabitol, potassium 2-ketogluconate, d-lactose, l-arabinose, dulcitol, *N*-acetylglucosamine, potassium 5-ketogluconate and d-turanose. Weak fermentation occurs from d-galactose, potassium gluconate, d-glucose, gentiobiose and d-lyxose. Amygdalin, arbutin, salicin and d-cellobiose provided positive results for one of three strains tested. The enzymatic activities present are F6PPK, α-glucosidase and *β*-galactosidase, but not lipase C14, *α*-chymotrypsin, *β*-glucuronidase, *α*-mannodisade, *α*-fucosidase, esterase lipase C8, esterase C4, naphthol-AS-BI-phosphohydrolase, alkaline phosphatase, trypsin, valine arylamidase and *N*-acetyl-*β*-glucosaminidase. Weak enzymatic activities are *β*-glucosidase, leucine arylamidase, cystine arylamidase, acid phosphatase and *α*-galactosidase.

The WK012_4_13^T^ (= LMG 33104^T^ = DSM 116073^T^), designated as the type strain, was obtained from a water kefir sample. The G+C DNA content of this strain is 56%. The GenBank accession numbers for the 16S rRNA gene and the whole-genome sequences are OR260337 and GCF_041080835.1, respectively.

## Description of *B*. *aquikefiricola *sp. nov.

*B. aquikefiricola* (a.qui.ke.fi.ri’co.la. L. fem. n. *aqua*, water; N.L. n. *kefir*, kefir; L. suff. *–cola*, inhabitant; N.L. n. *aquikefiricola*, water kefir inhabiting).

The cells are Gram-positive staining, non-motile, non-spore-forming, F6PPK-positive, catalase- and oxidase-negative, square rod shaped with an average length of 1.18±0.23 µm and an average width of 0.48±0.08 µm. The colonies are white and spherical after 48 h of incubation in MRScys. Growth occurs under anaerobic and aerophilic conditions. The strain grows in the temperature interval of 15–37 °C, the pH range of 4–8 and the NaCl range of 0–3%. Optimal conditions for growth occur at 30 °C and a pH value of 6. Fermentation occurred from d-ribose, d-fructose, aesculin ferric citrate, d-maltose, d-melibiose, d-raffinose, d-glucose and dulcitol but not from glycerol, erythritol, l-xylose, d-adonitol, methyl-*β*-d-xylopyranoside, l-sorbose, l-rhamnose, inositol, d-mannitol, d-sorbitol, methyl-*α*-d-mannopyranoside, d-trehalose, inulin, d-melezitose, starch, glycogen, xylitol, d-tagatose, d-fucose, l-fucose, d-arabitol, l-arabitol, potassium 2-ketogluconate, d-lactose, d-arabinose, d-xylose, l-arabinose, d-galactose, potassium gluconate, d-mannose, d-saccharose, *N*-acetylglucosamine, potassium 5-ketogluconate, amygdalin, arbutin, salicin, d-cellobiose, gentiobiose, d-turanose and d-lyxose. A weak fermentation occurs from methyl-*α*-d-glucopyranoside. The enzymatic activities are F6PPK, α-glucosidase and *N*-acetyl-*β*-glucosaminidase but not C14, α-chymotrypsin, β-glucuronidase, α-mannodisade, α-fucosidase, esterase lipase C8, esterase C4, naphthol-AS-BI-phosphohydrolase, alkaline phosphatase, trypsin, β-galactosidase and α-galactosidase. Weak enzymatic activities are valine arylamidase, β-glucosidase, leucine arylamidase, cystine arylamidase and acid phosphatase.

The WK041_4_12^T^ (= LMG 33105^T^ = DSM 116074^T^), designated as the type strain, was obtained from a water kefir sample. The G+C DNA content of this strain is 53.9%. The GenBank accession numbers for the 16S rRNA gene and the whole genome sequences are OR247709 and GCF_041080795.1, respectively.

## supplementary material

10.1099/ijsem.0.006549 Uncited Supplementary Material 1.

## Abbreviations

ANI: average nucleotide identity
BIC: bayesian information criterion
BTS: bacterial test standard
dDDH: digital DNA–DNA hybridization
F6PPK: fructose-6-phosphate phosphoketolase
HMDS: hexamethyldisilazane
LPSN: list of prokaryotic names with standing in nomenclature
MAG: metagenome-assembled genome
MSP: main spectra profile
OGRI: overall genome correlation index
ONT: Oxford Nanopore Technologies
SEM: scanning electron microscopy
