# Supplementary material for: Bifidobacterium fermentum sp. nov. and Bifidobacterium aquikefiricola sp. nov., isolated from water kefir
Source: Int J Syst Evol Microbiol. 2024 Oct 24;74(10):006549. doi: 10.1099/ijsem.0.006549 (PMC11500810; doi:10.1099/ijsem.0.006549)
Supplement: Uncited Supplementary Material 1. [file ijsem-74-06549-s001.pdf]

International Journal of Systematic and Evolutionary Microbiology

Supplementary material

Bifidobacterium fermentum sp. nov. and *Bifidobacterium aquikefiricola* sp. nov., isolated from water kefir.

Authors: Samuel Breselge[1,2]*, Paolo Bellassi[3]*, Coral Barcenilla[4], Avelino Álvarez-Ordóñez[4], Lorenzo Morelli[3], Paul D. Cotter[1,2,5#]

*these authors have contributed equally

#Corresponding author: All correspondence should be addressed to Paul D. Cotter: paul.cotter@teagasc.ie

[1]: Teagasc Food Research Centre, Moorepark, Cork, Ireland

[2]: APC Microbiome Ireland, Cork, Ireland

[3]: Department for Sustainable Food Process-DiSTAS, Università Cattolica del Sacro Cuore, Via Bissolati, 74, 26100 Cremona, Italy

[4]: Department of Food Hygiene and Technology, University of León, León, Spain

[5]: VistaMilk, Cork, Ireland

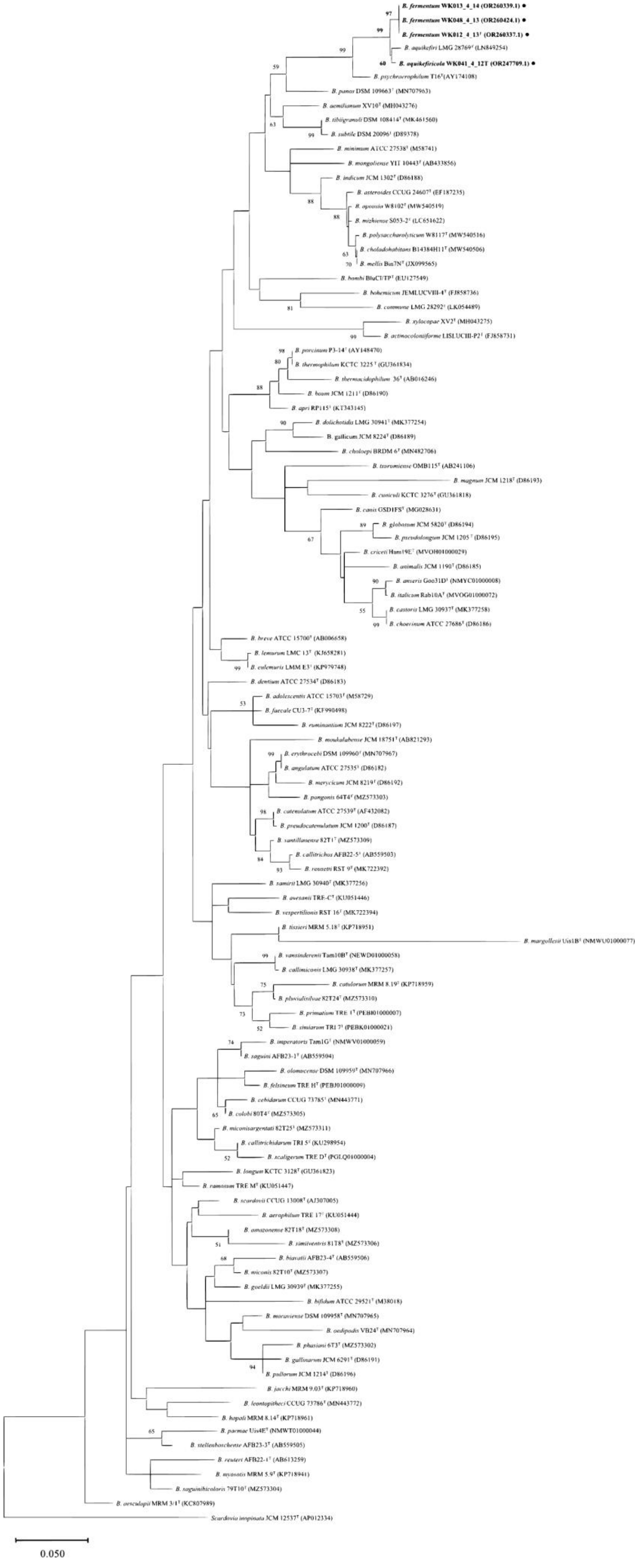

B. fermentum WK013_4_14 (OR260339.1) ●
B. fermentum WK048_4_13 (OR260424.1) ●
B. fermentum WK012_4_13^{T} (OR260337.1) ●
B. aquikefiri LMG 28769^{T} (LN849254)
B. aquikefiricola WK041_4_12T (OR247709.1) ●
B. psychraerophilum T16^{T} (AY174108)
B. panos DSM 109963^{T} (MN707963)
B. aemilianum XV10^{T} (MH043276)
B. tibiigranuli DSM 108414^{T} (MK461560)
B. subtile DSM 20096^{T} (D89378)
B. minimum ATCC 27538^{T} (M58741)
B. mongoliense YIT 10443^{T} (AB433856)
B. indicum JCM 1302^{T} (D86188)
B. asteroides CCUG 24607^{T} (EF187235)
B. apousia W8102^{T} (MW540519)
B. mizhiense S053-2^{T} (LC651622)
B. polysaccharolyticum W8117^{T} (MW540516)
B. choladohabitans B14384H11^{T} (MW540506)
B. mellis Bin7N^{T} (JX099565)
B. bombi BluCI/TP^{T} (EU127549)
B. bohemicum JEMLUCVIII-4^{T} (FJ858736)
B. commune LMG 28292^{T} (LK054489)
B. xylocopae XV2^{T} (MH043275)
B. actinocoloniiforme LISLUCIII-P2^{T} (FJ858731)
B. porcinum P3-14^{T} (AY148470)
B. thermophilum KCTC 3225^{T} (GU361834)
B. thermacidophilum 36^{T} (AB016246)
B. boum JCM 1211^{T} (D86190)
B. apri RP115^{T} (KT343145)
B. dolichotidis LMG 30941^{T} (MK377254)
B. gallicum JCM 8224^{T} (D86189)
B. choloepi BRDM 6^{T} (MN482706)
B. tsurumiense OMB115^{T} (AB241106)
B. magnum JCM 1218^{T} (D86193)
B. cuniculi KCTC 3276^{T} (GU361818)
B. canis GSD1FS^{T} (MG028631)
B. globosum JCM 5820^{T} (D86194)
B. pseudolongum JCM 1205^{T} (D86195)
B. criceti Ham19E^{T} (MVOH01000029)
B. animalis JCM 1190^{T} (D86185)
B. anseris Goo31D^{T} (NMYC01000008)
B. italicum Rab10A^{T} (MVOG01000072)
B. castoris LMG 30937^{T} (MK377258)
B. choerinum ATCC 27686^{T} (D86186)
B. breve ATCC 15700^{T} (AB006658)
B. lemurum LMC 13^{T} (KJ658281)
B. eulemuris LMM E3^{T} (KP979748)
B. dentium ATCC 27534^{T} (D86183)
B. adolescentis ATCC 15703^{T} (M58729)
B. faecale CU3-7^{T} (KF990498)
B. ruminantium JCM 8222^{T} (D86197)
B. moukalabense JCM 18751^{T} (AB821293)
B. erythrocebi DSM 109960^{T} (MN707967)
B. angulatum ATCC 27535^{T} (D86182)
B. merycicum JCM 8219^{T} (D86192)
B. pongonis 64T4^{T} (MZ573303)
B. catenulatum ATCC 27539^{T} (AF432082)
B. pseudocatenulatum JCM 1200^{T} (D86187)
B. santillanense 82T1^{T} (MZ573309)
B. callitrichos AFB22-5^{T} (AB559503)
B. rousetti RST 9^{T} (MK722392)
B. samirii LMG 30940^{T} (MK377256)
B. avesanii TRE-C^{T} (KU051446)
B. vespertilionis RST 16^{T} (MK722394)
B. tissieri MRM 5.18^{T} (KP718951)
B. margollesii Uis1B^{T} (NMWU01000077)
B. vansinderenii Tam10B^{T} (NEWD01000058)
B. callimiconis LMG 30938^{T} (MK377257)
B. catulorum MRM 8.19^{T} (KP718959)
B. pluvialitilvae 82T24^{T} (MZ573310)
B. primatium TRE 1^{T} (PEBI01000007)
B. simiarum TRI 7^{T} (PEBK01000021)
B. imperatoris Tam1G^{T} (NMWV01000059)
B. saguini AFB23-1^{T} (AB559504)
B. olomucense DSM 109959^{T} (MN707966)
B. felsineum TRE H^{T} (PEBJ01000009)
B. cebidarum CCUG 73785^{T} (MN443771)
B. colobi 80T4^{T} (MZ573305)
B. miconisargentati 82T25^{T} (MZ573311)
B. callitrichidarum TRI 5^{T} (KU298954)
B. scaligerum TRE D^{T} (PGLQ01000004)
B. longum KCTC 3128^{T} (GU361823)
B. ramosum TRE M^{T} (KU051447)
B. scardovii CCUG 13008^{T} (AJ307005)
B. aerophilum TRE 17^{T} (KU051444)
B. amazonense 82T18^{T} (MZ573308)
B. simiiventris 81T8^{T} (MZ573306)
B. biavatii AFB23-4^{T} (AB559506)
B. miconis 82T10^{T} (MZ573307)
B. goeldii LMG 30939^{T} (MK377255)
B. bifidum ATCC 29521^{T} (M38018)
B. moraviense DSM 109958^{T} (MN707965)
B. oedipodis VB24^{T} (MN707964)
B. phasiani 6T3^{T} (MZ573302)
B. gallinarum JCM 6291^{T} (D86191)
B. pullorum JCM 1214^{T} (D86196)
B. jacchi MRM 9.03^{T} (KP718960)
B. leontopitheci CCUG 73786^{T} (MN443772)
B. hapali MRM 8.14^{T} (KP718961)
B. parmae Uis4E^{T} (NMWT01000044)
B. stellenboschense AFB23-3^{T} (AB559505)
B. reuteri AFB22-1^{T} (AB613259)
B. myosotis MRM 5.9^{T} (KP718941)
B. saguinibicoloris 79T10^{T} (MZ573304)
B. aesculapii MRM 3/1^{T} (KC807989)
Scardovia inopinata JCM 12537^{T} (AP012334)
0.050

Figure S1 Phylogenetic tree of strains WK048_4_13, WK013_4_14, WK012_4_13^T, WK041_4_12^T and its relationship to other closely related species of the genus *Bifidobacterium*. Tamura 3-parameter model was used for this analysis. The tree nodes show the bootstrap values (>50%) obtained from 1000 replications. The scale bar, labelled 0.05, represents the number of substitutions per site. To ensure accuracy, all locations with gaps and missing data were excluded (complete deletion option). The analysis included a total of 106 nucleotide sequences, with 886 positions in the final dataset. The sequence of *Scardovia inopinata* JCM 12537^T was used as outgroup.

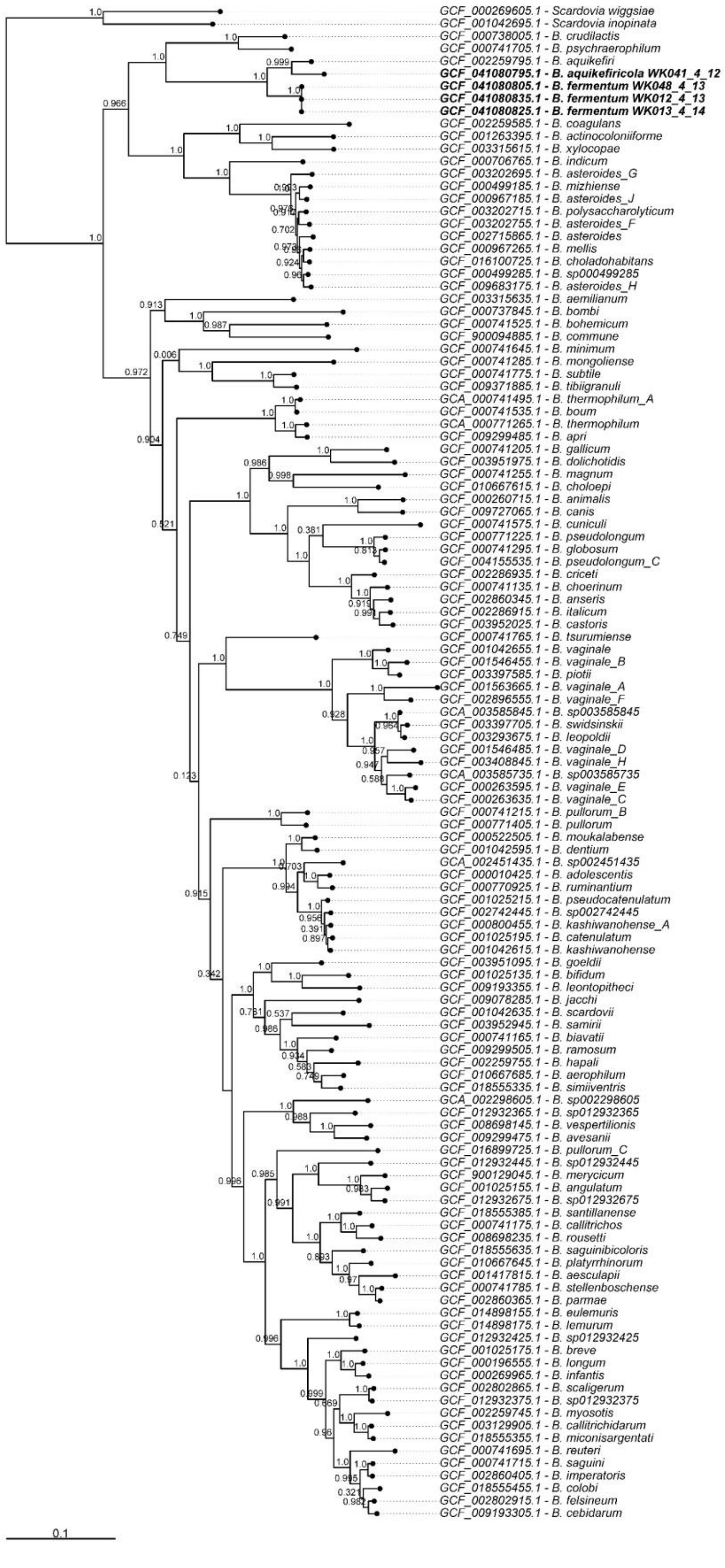

Figure S2: The marker gene based phylogenetic tree was inferred using the *de novo* workflow with previously published bifidobacteria genomes in GTDB-Tk. *Scardovia inopinata* JCM 12537T and *Scardovia wiggsie* F0424 was used as outgroup.

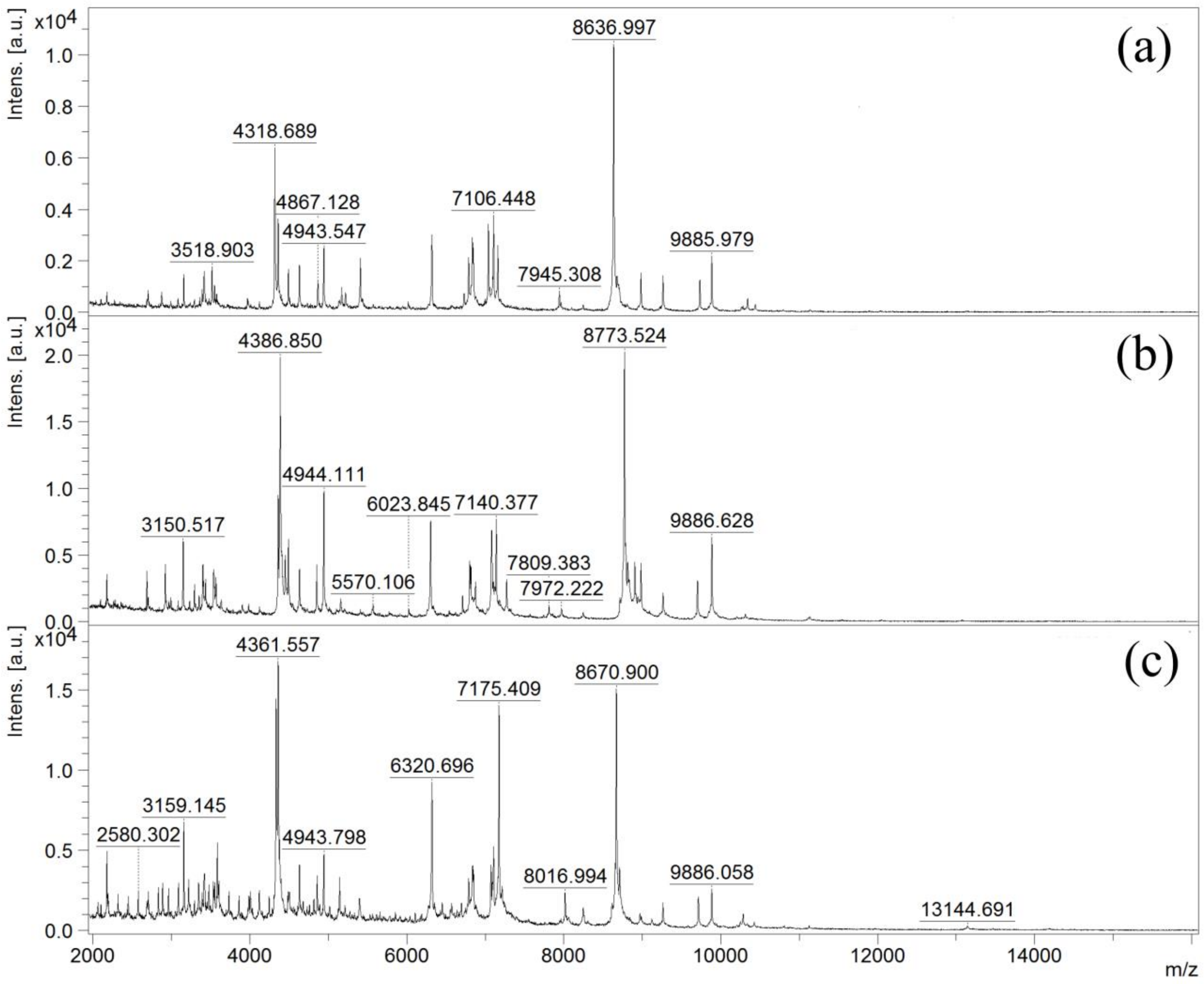

Figure S3 Comparison of the MALDI-TOF mass spectra of the chosen type strains of the two new candidate species, strain WK012_4_13^T (b) and strain WK041_4_12^T (c), with the closest species Bifidobacterium aquikefiri LMG 28769^T (a). The profile is shown in the range between 2,000 and 15,000 m/z and peak intensity is indicated in arbitrary units.
